# Mechanical and Functional Properties of Novel Biobased Poly(decylene-2,5-furanoate)/Carbon Nanotubes Nanocomposite Films

**DOI:** 10.3390/polym12112459

**Published:** 2020-10-23

**Authors:** Giulia Fredi, Andrea Dorigato, Mauro Bortolotti, Alessandro Pegoretti, Dimitrios N. Bikiaris

**Affiliations:** 1 Department of Industrial Engineering and INSTM Research Unit, University of Trento, Via Sommarive 9, 38123 Trento, Italy; andrea.dorigato@unitn.it (A.D.); mauro.bortolotti@unitn.it (M.B.); alessandro.pegoretti@unitn.it (A.P.); 2 Chemistry Department, Laboratory of Polymer Chemistry and Technology, Aristotle University of Thessaloniki, 54124 Thessaloniki, Greece; dbic@chem.auth.gr

**Keywords:** furan-based polyesters, carbon nanotubes, nanocomposites, mechanical properties, viscoelasticity, electrical properties

## Abstract

The present work investigates the microstructural, thermo-mechanical, and electrical properties of a promising, but still not thoroughly studied, biobased polymer, i.e., poly(decylene furanoate) (PDeF), and its performance when multi-walled carbon nanotubes (CNTs) are added. After sample preparation by solution mixing and film casting, the microstructural investigation evidences that the fracture surface becomes smoother and more homogeneous with a small fraction of CNTs, and that the production process is suitable to achieve good disentanglement and dispersion of CNTs within the matrix, although some aggregates are still observable. CNTs act as nucleating agents for PDeF crystals, as evidenced by differential scanning calorimetry, as the crystallinity degree increases from 43.2% of neat PDeF to 55.0% with a CNT content of 2 phr, while the crystallization temperature increases from 68.4 °C of PDeF to 91.7 °C of PDeF-CNT-2. A similar trend in crystallinity is confirmed by X-ray diffraction, after detailed Rietveld analysis with a three-phase model. CNTs also remarkably improve the mechanical performance of the bioderived polymer, as the elastic modulus increases up to 123% and the stress at break up to 131%. The strain at break also increases by +71% when a small amount of 0.25 phr of CNTs are added, which is probably the consequence of a more homogeneous microstructure. The long-term mechanical performance is also improved upon CNT addition, as the creep compliance decreases considerably, which was observed for both the elastic and the viscoelastic component. Finally, the films become electrically dissipative for a CNT content of 1 phr and conductive for a CNT amount of 2 phr. This study contributes to highlight the properties of bioderived furan-based polymer PDeF and evidences the potential of CNTs as a promising nanofiller for this matrix.

## 1. Introduction

The technological development in the last decades and increasing human activity have been lately associated with climate change, global warming, environmental pollution, fossil fuel depletion, and waste accumulation [1,2]. Therefore, one of the most interesting and urgent research questions and societal needs nowadays is to understand how to increase the efficiency of exploitation of renewable resources, to favor the transition toward a more sustainable and green future and alleviate the dependence of the modern economy on fossil-based resources [3,4,5].

Among the fossil-based products is also the vast majority of the most common synthetic polymers and plastics, which have become indispensable for many aspects of everyday life and industrial applications, thanks to their durability, versatility, processability, cost-effectiveness, and tailorable physical-mechanical properties [6]. The dichotomy between usefulness and environmental threat of plastic materials has directed considerable academic and industrial effort toward the research of more sustainable yet equally performing alternatives. A promising option is represented by the so-called bioplastics, i.e., plastics that are biodegradable and/or derived from renewable resources. More specifically, in the last decades, bio-derived and recyclable/compostable plastics have been the object of intense investigation, which brought on the market biopolymers like poly(lactic acid) (PLA) [7], polyhydroxyalkanoates (PHAs) [8], poly(butylene succinate) (PBS) [9], and thermoplastic starch [10,11]. The efficient obtainment of renewable monomers and the industrial-scale production of the deriving polymers have been in the spotlight of modern academic and industrial research [3]. Although bioplastics have been synthesized and investigated for almost a century, their intensive industrial production is still in its infancy. Global bioplastics production in 2019 was 2.11 million tons, only a small fraction compared to the 359 million tons of plastics produced yearly [12]. However, as the world is seeking a credible alternative to fossil-based plastics, the bioplastics market is expected to grow in the next years [12].

One of the most attractive bioderived monomers is furandicarboxylic acid (FDCA), obtainable from the fermentation and dehydration of biomass and listed among the twelve most important and strategic building blocks derived from renewable raw materials according to the US Department of Energy [13]. The attractiveness of FDCA derives from its chemical structure: it contains a heterocyclic furan ring, which confers stiffness to the molecule, and two di-acidic sites, suitable for the production of polymers via polycondensation [14]. Among the wide variety of polymers derivable from FDCA, furan-based polyesters or poly(alkylene furanoate)s (PAFs) are among the most important. Obtained through the polycondensation of FDCA with alkylene glycols, PAFs are emerging as a sustainable, bio-based alternative to fossil-derived poly(alkylene terephthalate)s (PATs), which dominate the market in the fields of packaging and synthetic fibers. PAFs have already demonstrated comparable or superior thermo-mechanical properties and superior gas barrier properties than those of the corresponding PATs, which make them suitable for packaging applications [14,15,16,17,18]. The most prominent members of this polymer family are poly(ethylene furanoate) (PEF) and poly(buthylene furanoate) (PBF), extensively researched as they are the bio-based alternative to poly(ethylene terephthalate) (PET) and poly(buthylene terephthalate) (PBT) [19,20], respectively. However, other furan-based polyesters have been produced by combining FDCA with longer-chain diols, containing up to 12 carbon atoms [17]. As shown in previous studies, furan-based polymers with short alkyl chains often have slow crystallization kinetics, and an annealing treatment is needed to produce a semi-crystalline material with relevant gas barrier properties [21]. This is partly mitigated in FDCA-based polyesters with longer alkyl chains, as the higher molecular mobility helps the local ordered arrangement of the polymer chains, thereby favoring the obtainment of a relevant crystallinity degree [17]. This is desirable for the final applications, as a higher crystallinity degree is generally associated with a higher gas-barrier performance [22]. Moreover, an increased number of carbon atoms in the monomer diol leads to mitigation of the thermo-mechanical properties, i.e., a decrease in the elastic modulus, glass transition temperature, and melting temperature and an increase in flexibility. For example, Tsanaktsis et al. [21] reported the synthesis of poly(decylene furanoate) (PDeF), obtained by combining FDCA with 1,10-decanediol, and performed a detailed microstructural and thermal investigation. PDeF showed a melting temperature of 112 °C, a glass transition temperature of 1 °C, and a tensile strength and elastic modulus similar to those of low-density polyethylene (LDPE).

One of the well-known ways to tailor the physical and mechanical properties of polymers is to add fillers and nanofillers. Polymer nanocomposites have attracted great attention in the last decades and opened the way for new materials with enhanced mechanical and functional properties. More specifically, there has been increasing interest and research on carbon-based nanofillers, such as carbon nanotubes (CNTs), expanded graphite nanoplatelets (xGnPs), graphene oxide (GO) and reduced graphene oxide (rGO), carbon nanofibers (CNF), and nanospheres (CNS). Such nanofillers confer enhanced thermal and mechanical properties, electrical and thermal conductivity, anti-static properties, self-sensing and self-healing abilities, and shape stabilization of phase-change materials for thermal energy storage [23,24,25].

Despite the interest in FDCA-based polyesters, there is a limited amount of information on the development of furan-based nanocomposites. The existing studies mainly focus on the addition of carbon-based nanofillers and nanoclays in PEF [15,26,27,28,29,30], but some research also deals with PBF [31], poly(propylene furanoate) (PPF) [32,33], or poly(hexamethylene furanoate) (PHF) [22], mainly prepared by in-situ polymerization in presence of a nanofiller suspension. In these cases, nanofillers are added to increase the mechanical properties but especially the crystallization kinetics and the gas barrier performance [22]. Conversely, to the best of the authors’ knowledge, no research can be found on the development of nanocomposites with FDCA-based polymers with longer alkyl chains. Moreover, only an exiguous number of studies can be found about the preparation of FDCA-based nanocomposites starting from an already polymerized polymer matrix.

This study reports for the first time the preparation and characterization of PDeF-based nanocomposites containing CNTs in various percentages. The nanocomposites were prepared from fully polymerized PDeF and commercial multi-walled CNTs (MW-CNTs), through solution mixing and solution casting techniques. This nanofiller was added to increase the mechanical properties, enhance the crystallinity degree (to improve the gas-barrier properties,) and decrease the electrical resistivity (to confer conductive or antistatic properties). The study initially targets and discusses the main challenges of this research, namely the solubility of PDeF in common organic solvent, the preparation of a stable CNT suspension, and the preparation of homogeneous PDeF films with a proper dispersion of this nanofiller. Subsequently, the results of the characterization of the prepared films, from the microstructural, thermal, short- and long-time mechanical, dynamic-mechanical, and electrical point of view, are presented.

## 2. Materials and Methods

### 2.1. Materials

Poly(decylene-2,5-furanoate) (PDeF) was synthesized by applying a variation of the two-stage melt polycondensation method (esterification and polycondensation) in a glass batch reactor as described in our previous work [21]. In brief, dimethyl 2,5-furandicarboxylate (DMFD) and 1,10-decanediol at a molar ratio of diester/diol = 1/2 were charged into the reaction tube of the polyesterification apparatus with 400 ppm tetrabutyl titanate (TBT). The reaction mixture was heated at 150 °C under argon atmosphere for 2 h, at 160 °C for an additional 2 h, and finally at 170 °C for 1 h. This first step (transesterification) is considered as completed after the collection of almost all the theoretical amount of CH_3_OH, which was removed from the reaction mixture by distillation and collected in a graduate cylinder. After this stage, the corresponding bishydroxydecylene-2,5-furan carboxylate monomers were formed. In the second stage, these monomers reacted with DMFD in a molar ratio 1/1.05 at 150 °C under argon atmosphere for 2 h, at 160 °C for an additional 2 h, and finally at 170 °C for 1 h. During this stage, methanol was also removed as a by-product. After that time, in the third step of polycondensation, a vacuum (5.0 Pa) was applied slowly over a period of time of about 30 min. The temperature was increased to 210 °C and the polymerization was continued for 1 h at this temperature, followed by 220 °C for 1 h and 230 °C for 0.5 h, at a stirring speed 720 rpm. After the polycondensation reaction was completed, the polyester was easily removed, milled, and washed with methanol. The intrinsic viscosity of the produced polyester was measured with an Ubbelohde viscometer (Schott Gerate GMBH, Hofheim, Germany) at 25 °C in a phenol and tetrachloroethane (60/40, *w*/*w*) mixture. In order to achieve complete dissolution, the sample was heated in the solvent mixture at 80 °C for 20 min. After cooling, the solution was filtered through a disposable Teflon filter. The calculation of the intrinsic viscosity [η] of the polymer was based on the Solomon-Ciuta equation (Equation (1)) of a single point measurement [34,35]:

(1) $$\left[\eta \right]=\frac{{\left[2(\frac{t}{t_{0}}-\ln\left(\frac{t}{t_{0}}\right)-1)\right]}^{\frac{1}{2}}}{c}$$

where c is the concentration of the polymer solution.

The obtained intrinsic viscosity value of PDeF is [η] = 0.68 dL/g. The number average molecular weight ($M_{n}$) of the PDeF polyester was calculated from the intrinsic viscosity [η] values, using the Berkowitz equation (Equation (2)) as was modified in our previous work [36,37]:

(2) $$M_{n}=3.29\times 10^{4}{\left[\eta \right]}^{1.54}$$

The obtained molecular weight of PDeF polyester is $M_{n}$= 18,166 g/mol.

Multi-walled CNTs Nanocyl NC7000 were purchased from Nanocyl SA (Sambreville, Belgium). Chloroform (HPLC grade) was purchased from Fisher Chemicals (Thermo Fisher Scientific Inc., Waltham, MA, USA). Hexafluoro-2-propanol (HFIP) (purity 99%) was purchased from Carlo Erba Reagents S.r.l. (Milano, Italy). Dimethylformamide (analytical reagent) was purchased from RCI Labscan Ltd. (Bangkok, Thailand). All the materials were used as received.

### 2.2. Sample Preparation

PDeF-based nanocomposites were prepared via solvent casting. PDeF was dissolved in a mixture of chloroform and HFIP (9:1 vol:vol), as this solvent mixture has been reported to dissolve polyesters in general and furan-based polyesters in particular [38]. The polymer concentration in the solvent was 1 g of polymer in 25 mL of solvent, and the solutions were magnetically stirred at 40 °C for 1 h. Meanwhile, CNTs were sieved with a 100 µm metallic sieve to remove large aggregates and ultrasonicated for 15 min in DMF (0.5 mg/mL) with an ultrasonic tip (UP-400S, Hielscher Ultrasonics GmbH, Teltow, Germany). The CNT suspension was added to the PDeF solution and the mixture was magnetically stirred at 40 °C for 2 h, mildly ultrasonicated for 10 min in a ultrasonic bath Labsonic LBS1 (Falc Instruments s.r.l., Bergamo, Italy), casted in a Petri dish, and left for 24 h at room temperature and 3 h at 50 °C. The process led to the production of free-standing nanocomposite films with different CNT concentrations (0/0.25/0.5/1/2 parts per hundred resin (phr)) and a thickness of 50–80 µm. The obtained nanocomposite films were stored in a desiccator with dry silica salts until use. Table 1 lists the prepared samples with nominal weight compositions.

### 2.3. Experimental Techniques

Optical microscope (OM) micrographs of the prepared films were obtained with a Wild Heerbrugg M3Z optical microscope (Heerbrugg, Switzerland) equipped with an Allied Pike F032C camera (Allied Vision Technologies GmbH, Exton, PA, USA). SEM micrographs of the cryo-fracture surfaces of the prepared films were obtained with an FE-SEM Zeiss Supra 60 (Carl Zeiss AG, Oberkochen, Germany) at different magnification levels, after Pt-Pd sputtering. 

Fourier-transform infrared (FTIR) spectroscopy was carried out in attenuated total reflectance (ATR) mode with a Perkin-Elmer Spectrum One instrument (Perkin Elmer GmbH, Waltham, MA, USA). Data were collected in the wavenumber range 650–4000 cm^−1^, and four scans were superimposed for each spectrum (resolution 4 cm^−1^).

X-ray diffraction measurements were performed on a Italstructures IPD3000 instrument equipped with a cobalt anode X-ray source (Co_kα_ = 1.788965 Å) operating at 40 kV, 20 mA, coupled to an incident beam multilayer monochromator, 1° divergence slit, and 5° Soller slits. Specimens of about 10 × 5 mm were fixed on a zero-background Si sample holder and positioned in reflection geometry; diffraction patterns were acquired by means of a Inel CPS120 curved position-sensitive detector, over the 10°–70° (2*θ*) range with a 0.03° channel resolution and 3600 s total counting time. Instrumental broadening was characterized by means of an Y_2_O_3_ powder (99.99%, Sigma-Aldrich, St. Louis, MO, USA, CAS# 1314-36-9) annealed at 1400 °C for 24 h.

Differential scanning calorimetry (DSC) was performed with a Mettler DSC 30 calorimeter (Mettler Toledo, Inc., Columbus, OH, USA), at 10 °C/min, between –50 and 250 °C, under a nitrogen flow of 100 mL/min. Specimens of approx. 5 mg were sealed in aluminum crucibles and subjected to a first heating scan, a cooling scan, and a second heating scan. The test allowed the measurement of the glass transition temperature ($T_{g}$), the melting and crystallization temperatures ($T_{m}$, $T_{c}$), and the enthalpy values ($∆H_{m}$, $∆H_{c}$) of the polymer phase. One specimen was tested per composition. The temperatures were measured with a precision of 0.02 °C and the enthalpies with a precision of 1% and an accuracy of 4%.

Thermogravimetric analysis (TGA) was carried out with a Q5000IR thermobalance (TA Instruments, Inc., New Castle, DE, USA). Specimens of approx. 4 mg were tested at a heating rate of 10 °C/min up to 700 °C, under a nitrogen flow of 10 mL/min. The test allowed the measurement of the temperatures corresponding to mass losses of 1 wt%, 3 wt%, and 5 wt% ($T_{1\%}$, $T_{3\%},\;T_{5\%}$), and the degradation temperature ($T_{d}$), intended as the peak of the mass loss derivative signal and corresponding to the temperature at the maximum degradation kinetics.

Dynamic-mechanical analysis (DMA) was performed with the TA Instruments DMA Q800 equipped with a 16 N load cell. Rectangular specimens with nominal in-plane dimensions of 30 × 5 mm^2^ were cut out of the prepared films and mounted on the instrument with a gauge length of 10 mm (calculated as the distance between the grips). Tests were performed in tensile mode between –50 and 80 °C, at 3 °C/min, with a strain amplitude of 0.05% applied at a frequency of 1 Hz.

Quasi-static tensile tests were performed at room temperature with the same instrument and the same sample geometry was described for a DMA test. At least four specimens for each composition were tested at a crosshead speed of 100 µm/min.

Creep-recovery tests were performed at 30 °C with the same instrument, sample geometry, and configuration described for DMA tests. A constant stress of 1 MPa was applied for 60 min and the recorded displacement was used to calculate the creep compliance as a function of time (D(t)), determined as the ratio between the measured strain and the constant stress. The stress was then removed and the specimen was left recovering for 60 min, while recording the displacement.

Finally, electrical resistivity was measured to assess the anti-static properties of the prepared films. The measurement was performed in a four-point configuration, according to the standard ASTM D4496-04, on rectangular specimens with in-plane dimensions of 10 × 50 mm^2^. A voltage generator ISO-Tech IPS 303DD (Milano, Italy) was connected to the specimen, an ammeter was connected in series to measure the flowing current, and a voltmeter was connected to the two inner electrodes to measure the voltage drop. The volume resistivity ρ (Ω·cm) was measured through Equation (3):

(3) $$\rho =R\frac{w\cdot t}{l}$$

where R is the resistance calculated as the slope of the voltage–current plot, linear in the measurement range, w and t are the specimen width and the thickness, respectively, and l is the distance between the inner electrodes, constant and equal to 3.69 mm. This configuration allows the measurement of values of resistivity up to 10^7^ Ω·cm, while the resistivity of more insulating samples was measured according to ASTM D257 using a Keithley 6517A electrometer/high-resistance meter (Cleveland, OH, USA) and an 8009 resistivity test fixture at room temperature. In this test, a constant voltage of 50 V was applied to square samples with an in-plane area of 64 × 64 mm^2^.

## 3. Results and Discussion

### 3.1. Microstructural Properties

**Figure 1:**
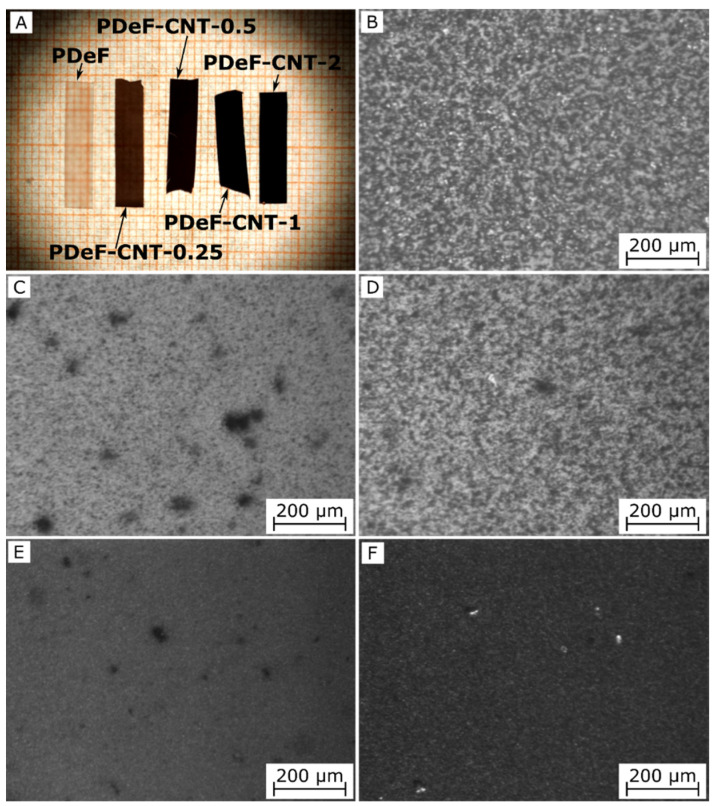
Overview and optical microscope (OM) micrographs of the prepared films. (**A**) Overview on graph paper (thick lines = square centimeters; thin lines = square millimeters); (**B**) poly(decylene furanoate) (PDeF); (**C**) PDeF-CNT-0.25; (**D**) PDeF-CNT-0.5; (**E**) PDeF-CNT-1; (**F**) PDeF-CNT-2.

**Figure 2:**
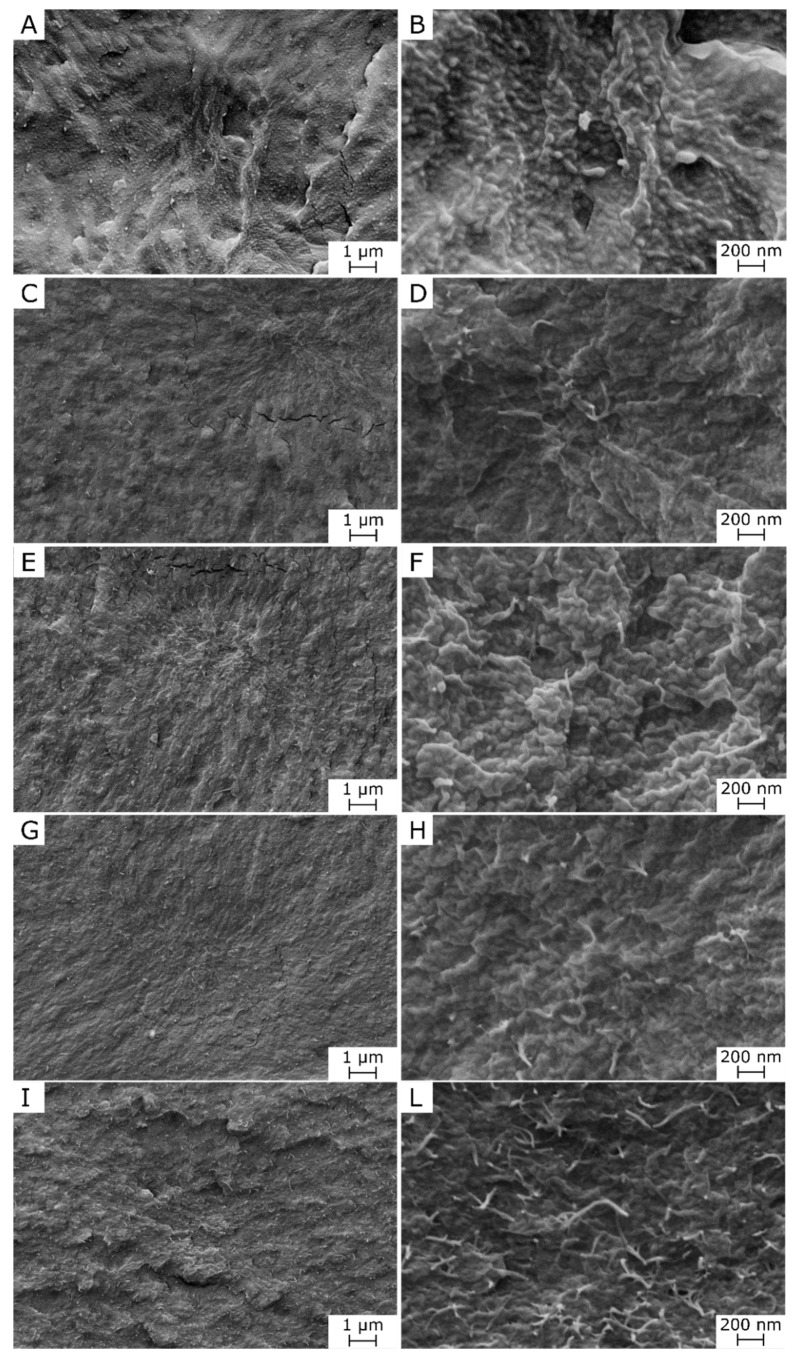
Scanning electron microscopy (SEM) micrographs of the cryofracture surface of the prepared films, at two different magnification levels. (**A**,**B**) PDeF; (**C**,**D**) PDeF-CNT-0.25; (**E**,**F**) PDeF-CNT-0.5; (**G**,**H**) PDeF-CNT-1; (**I**,**L**) PDeF-CNT-2.

Figure 1 show an overview and optical microscope micrographs of the prepared films. The neat PDeF appears white and translucent, and an addition of CNTs progressively alters the color and transparency of the films. From the OM micrographs, it is evident that the neat PDeF film (Figure 1B) shows a semicrystalline structure with coarse grains, while the sample PDeF-CNT-0.25 (Figure 1C) shows a more uniform microstructure with CNTs generally well-dispersed, but some bigger aggregates (10–20 µm) are present. A similar consideration can be done for the specimens with a higher concentration of CNTs (Figure 1D–F). An improvement in the microstructural homogenization with CNT addition is observable also from SEM micrographs of the cryofracture surface of the prepared films, reported in Figure 2A–L. From the low-magnification micrographs, Figure 2A,C,E,G,I, it is clear that the fracture surface becomes smoother and more homogeneous by adding CNTs, and this is particularly evident by comparing the neat PDeF (Figure 2A) and the sample PDeF-CNT-0.25 (Figure 2C). The high-magnification micrographs show the fracture morphology with single nanotubes emerging from the fracture surface, observable especially from the micrograph of the sample PDeF-CNT-2 (Figure 2L). Therefore, the processing route of these polymer nanocomposites is suitable to disentangle pristine multi-walled CNTs and to achieve an acceptable dispersion of CNTs in a polymer matrix, which can be difficult for solvent-based preparations, as they involve lower shear stresses than those found in the processing routes with polymer melts.

**Figure 3:**
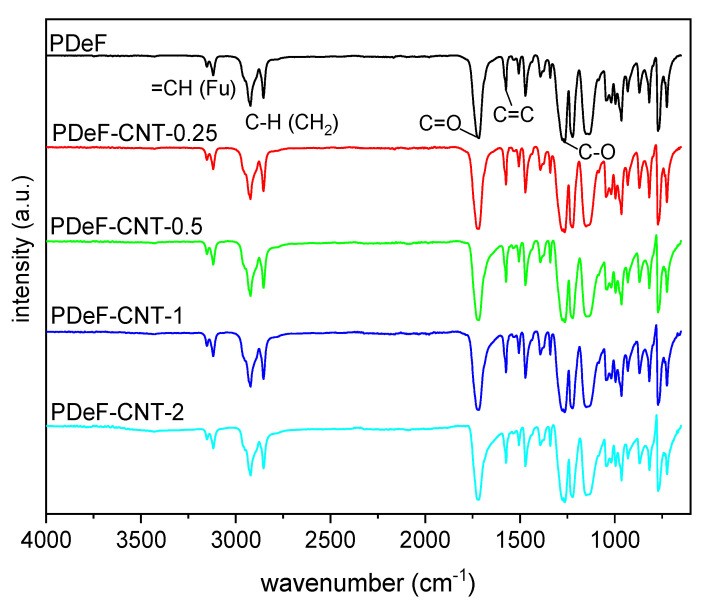
Attenuated total reflectance Fourier-transform infrared (ATR-FTIR) spectra (baseline-corrected and vertically translated) of the prepared samples (Fu = furan ring).

Figure 3 shows the attenuated total reflectance Fourier-transform infrared (ATR-FTIR) spectra of the prepared nanocomposite films. Spectra were baseline-corrected, normalized to the peak with the highest intensity (located at 1720 cm^−1^), and vertically shifted to facilitate their comprehension. The spectra of neat PDeF are consistent with the macromolecular structure of this polymer, which can be confirmed by the symmetrical and asymmetrical stretching of the furan ring at 3119 and 3152 cm^−1^, respectively, the symmetrical and asymmetrical C–H stretching characteristics of the methylene groups of the alkyl chain at 2920 and 2850 cm^−1^, respectively, the vibration of the C=C bond of furan at 1574 and 1530 cm^−1^, and a very intense band at 1718 cm^−1^, arising from the carbonyl stretching vibration C=O, typical of ester groups [5,39]. The furan ring breathing can also be observed at 1018 cm^−1^ and ring bending at 965, 817, and 772 cm^−1^ [40]. Moreover, the absorption peak of −OH (approx. 3400 cm^−1^) is not observable, which indicates that the employed polymer has a relatively high molecular weight and the prepared film has been properly stored in dry conditions [41]. The spectra of the nanocomposites show the same peaks observed for the neat PDeF, without red- or blue-shifts or evident changes in the relative intensity of the peaks. This indicates that the formation of covalent bonds between the polymer chains and the fillers is not observed, as expected. The only evidence of the presence of CNTs is observable in the spectrum of PDeF-CNT-2 as a broad and low-intensity peak at 3400 cm^−1^, which derives from the -OH vibration generally present on the surface of commercial CNTs [42]. In any case, it is difficult to observe the FTIR vibrations of carbon-based materials, and CNTs in particular, through an ATR technique.

**Figure 4:**
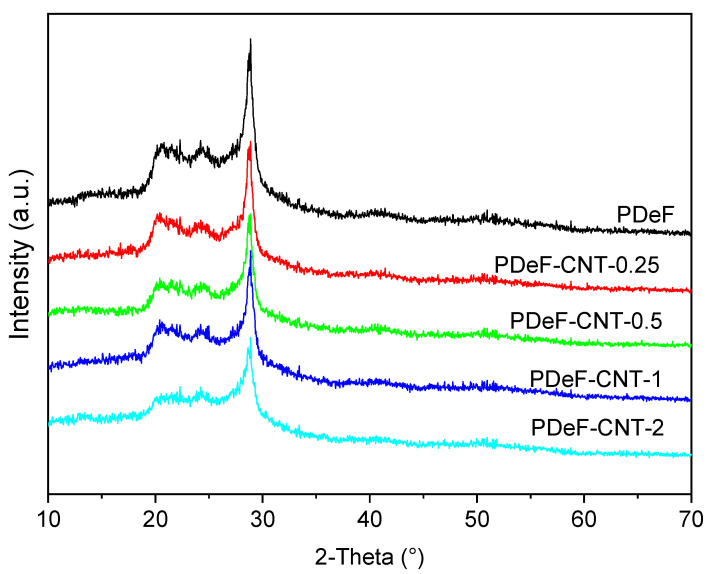
X-ray diffraction data acquired on the prepared samples.

Figure 4 shows the X-ray diffraction data acquired on the prepared samples. Diffraction patterns exhibit the typical feature of semicrystalline materials, with sharp Bragg reflections located at 20.5°, 24.2°, and 28.8° (for Co_kα_ radiation) and a diffuse background arising from the amorphous fraction. Additionally, it is possible to qualitatively observe a progressive increase in the peak tails (Lorentzian broadening) of the main reflection at 28.8° with rising CNT content; as discussed later, this effect can be associated with modifications in the size distribution of the coherently scattering domains.

As reported in [43], although no detailed crystallographic characterization has been carried out on the PDeF structure, its powder pattern closely resembles those of aromatic–aliphatic polyesters with long methylene chains in the diol units; it is thus reasonable to assume a triclinic β–form as a starting point for the microstructural analysis and the crystalline fraction evaluation (see also [21]).

**Figure 5:**
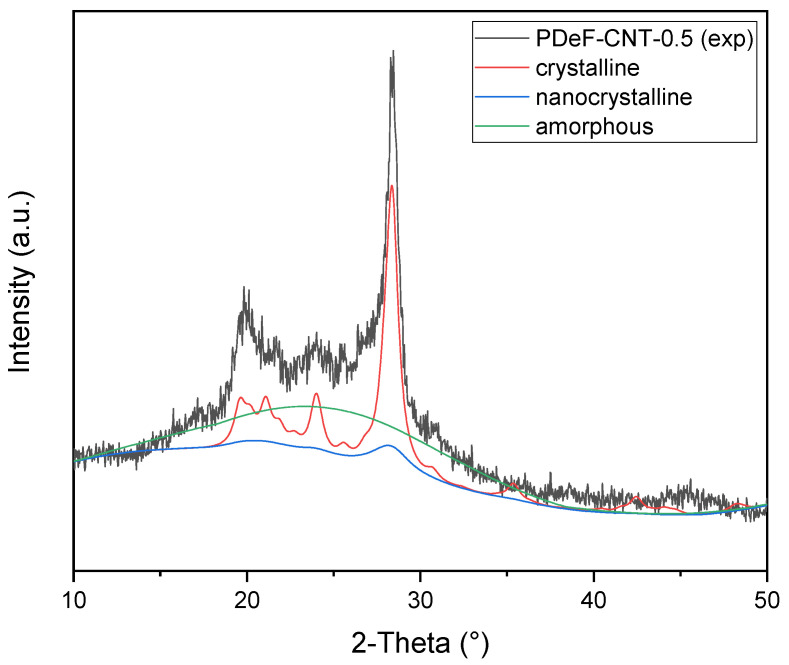
Rietveld modeling of diffraction data showing the deconvolution of the three phase contributions, namely crystalline, nanocrystalline, and amorphous PDeF fractions.

A full-profile modeling of the diffraction trace was carried out by means of the Maud Rietveld software [44] to assess in a semi-quantitative manner the degree of crystalline ordering. A three-phase model was adopted, taking into account not only the amorphous and crystalline fractions but also an additional nanocrystalline (or paracrystalline) phase, to better account for the Lorentzian peak tails; all the phases shared the same crystallographic structures with different average domain sizes to account for the various broadenings in the pattern. The initial analysis was carried out on the neat PDeF sample, refining triclinic lattice constants, volume fractions of all the phases, as well as the average crystallite dimensions of the crystalline and nanocrystalline fractions by adopting the model described in Chapter 8 of [45]. These parameters refined to average values of about 85 and 25 Å, respectively, and were successively fixed for the CNT-containing samples; the domain size of the amorphous fraction was set to 10 Å, as a best fit to the diffuse halo above the background baseline. An example fit for PDeF-CNT-0.5 is reported in Figure 5. It is worth noting that the introduction of two crystalline phases with different, fixed average domain sizes represents an approximation with respect to the real microstructure, which could be better described with a distribution of domain sizes and different types of lattice disorder (e.g., dislocations, strains). However, it gives a reasonably accurate description of reflection broadening and is, thus, acceptable for the aim of a semi-quantitative evaluation of the crystallinity degree.

Table 2 reports the results from Rietveld analysis, including the final $R_{wp}$ figure of merit, volume fractions of the three phases, as well as the total degree of crystallinity ($X_{c}$). The same table also presents, for the sake of comparison, the crystallinity degree obtained using the approximated Equation (4):

(4) $$X_{c}=\frac{I_{c}}{I_{c}+I_{a}}$$

where $I_{a}$ is the integrated intensity of the amorphous diffuse signal and $I_{c}$ are the total integrated intensities of the Bragg peaks, as described, e.g., in [46]. This latter quantification approach typically underestimates the total crystalline volume fraction, due to the inability to separate overlapping peaks and correctly model the diffuse signal arising from the paracrystalline fraction; despite the numerical discrepancies, both methods point to a general rise in the degree of crystallinity with the increase in the CNT content, which agrees with DSC results (see Section 3.2).

Interestingly, the Rietveld analysis gives further details about the nature of the crystalline domains, showing an increase in the macrocrystalline fraction at the expense of the nanocrystalline one in the PDeF-CNT-0.25 and PDeF-CNT-0.5 samples, with an opposite trend in PDeF-CNT-1 and PDeF-CNT-2; this could hint at an initial rise in the average crystalline domains size for low-CNT-content samples, which is then reversed for higher CNT contents.

### 3.2. Thermal and Dynamic-Mechanical Properties

**Figure 6:**
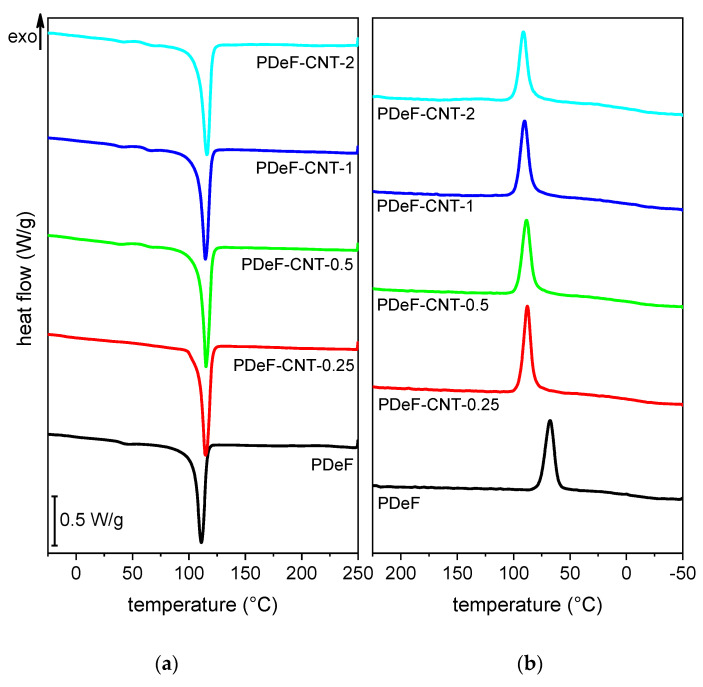
Differential scanning calorimetry (DSC) thermograms of the prepared samples. (**a**) First heating scan and (**b**) cooling scan.

Figure 6 shows the DSC thermograms of the prepared samples, while the most important DSC results are presented in Table 3. The neat PDeF sample shows an endothermic melting peak at 110.2 °C and a glass transition temperature of −4 °C, in good agreement with the analysis performed by Jiang et al. [43] and Tsanaktsis et al. [21]. It is interesting to observe that the melting temperature is very close to that of LDPE (110–115 °C), a material widely used for packaging applications. The addition of CNTs shifts the melting peak to higher temperatures and increases the melting enthalpy (measured in the first heating scan) from 66.2 J/g of the neat PDeF to 82.6 J/g of the sample PDeF-CNT-2. From these enthalpy values, the crystallinity degree ${Χ}_{c}$ was calculated through Equation (5):

(5) $${Χ}_{c}=\frac{∆H_{m}}{\omega_{p}\cdot ∆H_{m,0}}\cdot 100$$

where $∆H_{m}$ is the melting enthalpy measured on each sample, $\omega_{p}$ is the weight fraction of PDeF in each sample, and $∆H_{m,0}$ is the enthalpy of a fully crystalline PDeF, calculated as 153 J/g by Tsanaktsis et al. [21]. As reported in Table 3, the crystallinity degree increases considerably with the CNT fraction, as CNTs act as nucleating agents for the crystallization of the polymer matrix during solvent evaporation but also during solidification of a melt. This effect is commonly reported in the literature for a wide variety of polymer matrices containing nanofillers, especially with CNTs [4,22,26,28,30,39,47]. The values of crystallinity degree calculated with DSC results are slightly lower than those determined by XRD, but the results are of the same order of magnitude and show the same trend, which confirms the reliability of the XRD analysis and the calculated value of $∆H_{m,0}.$

The nucleating effect can also be observed in the cooling scan. The crystallization peak for the neat PDeF is found at 68.4 °C and the crystallization enthalpy is 49.4 J/g. With CNT addition, the crystallization peak shifts to higher temperatures (up to 91.7 °C) and the enthalpy increases (up to 57.7 J/g), which both indicate that CNTs act as nucleating agents also during the solidification from the melted phase. The second heating scan shows the same characteristics as the first, as CNTs increase the melting temperature and enthalpy. However, the enthalpy values are lower than those of the first scan, which indicates that a scanning speed of 10 °C/min applied during DSC tests is not enough to obtain the maximum crystallinity degree of these samples, for which the annealing phase is a necessary step.

The glass transition temperature, measured in the second heating scan, is also reported in Table 3. The values of $T_{g}$ vary in the range −10.8/−2 °C, in good agreement with data from the literature [21,43], and do not follow a trend with the CNT fraction. However, it is difficult to precisely locate the $T_{g}$ as the signal is very weak. Therefore, a more accurate analysis was carried out with DMA, as reported hereafter (Section 3.2).

**Figure 7:**
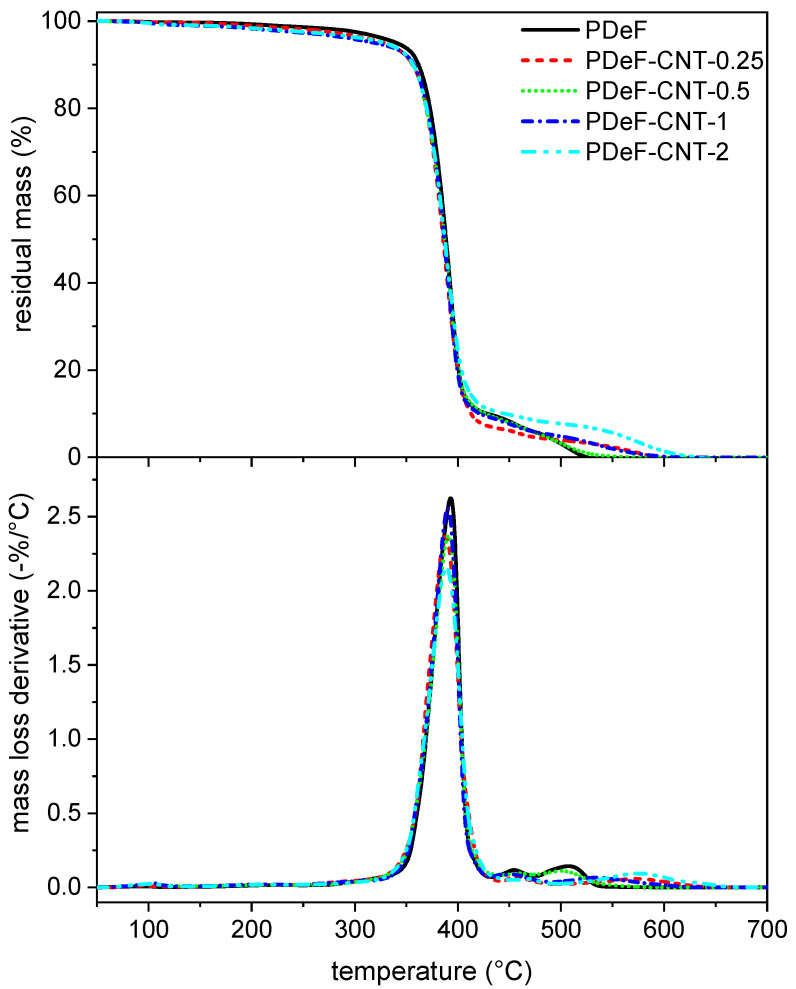
Thermogravimetric analysis (TGA) thermograms of the prepared samples. Residual mass (top) and mass loss derivative as a function of temperature.

Figure 7 shows the TGA thermograms of the prepared films, while the most important TGA results are collected in Table 4.

The neat PDeF loses the majority of its mass between 350 °C and 500 °C. It shows a mass loss of 5 wt% at 340.4 °C, and the mass loss derivative peak occurs at 393 °C, in agreement with results from the literature [21]. The addition of CNTs does not substantially modify the mass loss trend, but the temperatures corresponding to the beginning of the degradation ($T_{1\%}$ and $T_{3\%}$) are significantly decreased, especially for a CNT content above 0.5 phr, which can be due to a small amount of residual atmospheric water that the samples could have absorbed before the test. On the other hand, $T_{5\%}$ and $T_{d}$ show a small variation after CNT addition (probably due to small variations in the specimen shapes or masses), which indicates that the thermal degradation behavior is generally not affected by the CNT content.

**Figure 8:**
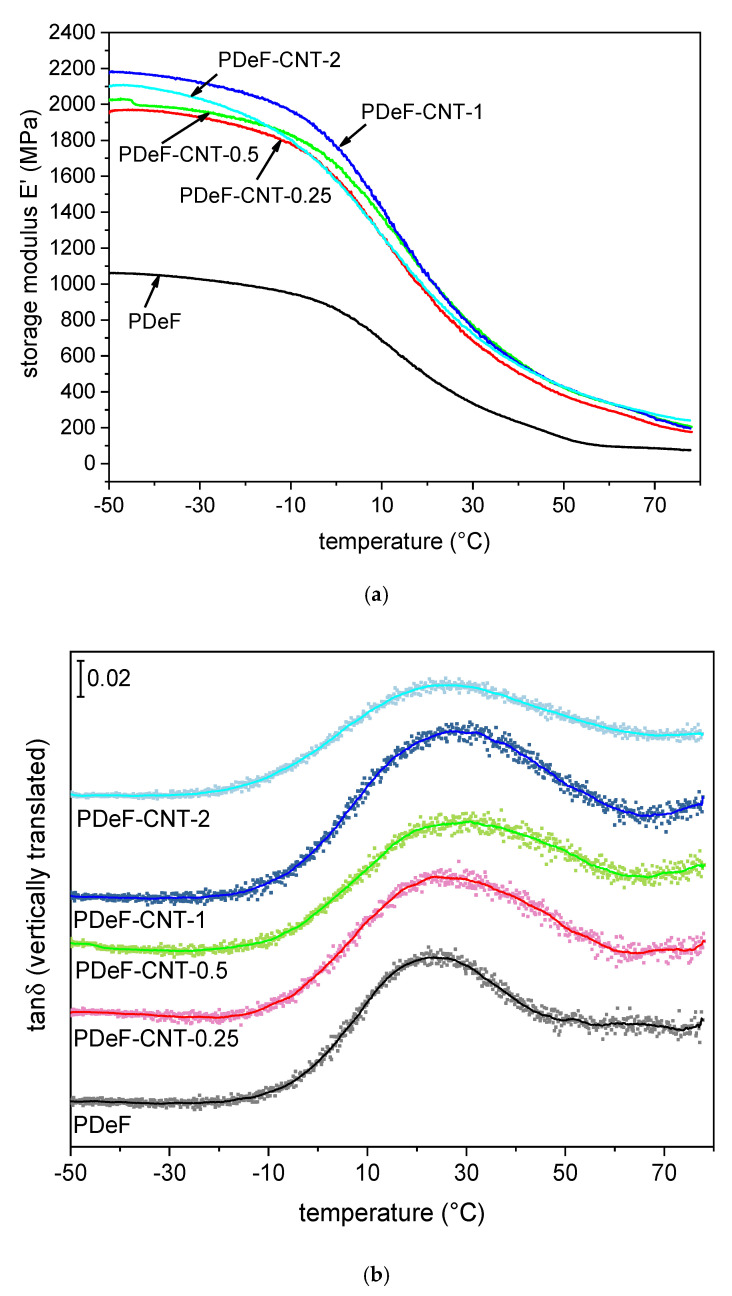

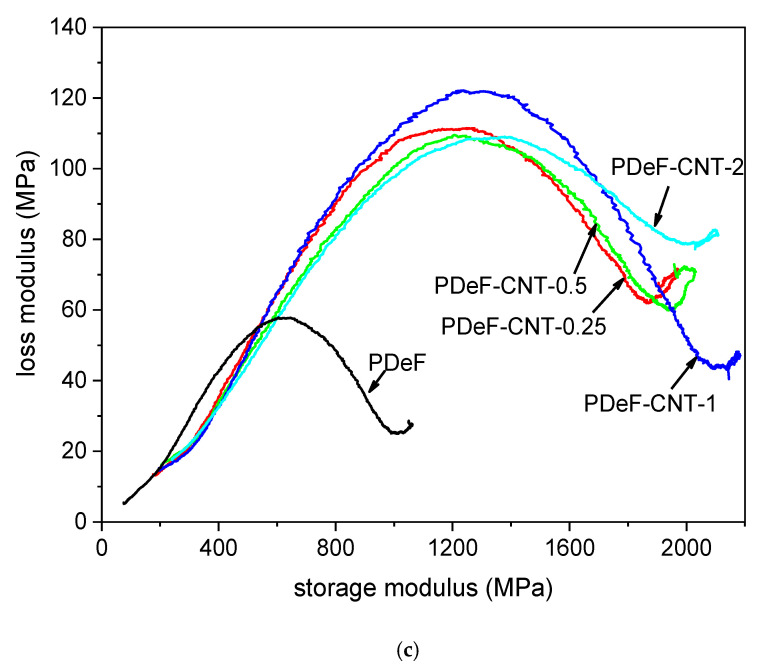
Results of dynamic-mechanical analysis (DMA). (**a**) Storage modulus as a function of temperature; (**b**) tan *δ* (vertically translated, experimental data with dots and smoothed curves with solid lines) as a function of temperature; (**c**) Cole–Cole plot (smoothed), reporting the loss modulus *E”* as a function of the storage modulus *E’*.

Another important test that can be done on polymer films is DMA, which gives information on the trend of the viscoelastic parameters storage modulus (*E*’), loss modulus (*E*”), and loss tangent (*tan*δ), as a function of temperature. The results of the DMA tests are reported in Figure 8 and Table 5. Figure 8a shows the trends of the storage modulus as a function of temperature. For the neat PDeF, the storage modulus is approx. 1140 MPa at −50 °C, and it decreases with temperature. A step in E’ is found while approaching the glass transition temperature interval, which ranges from 0 °C to 40 °C. A similar behavior can be observed for the CNT-enhanced nanocomposites, but the values of *E*’ are remarkably higher than those of the neat polymer film. For example, the sample PDeF-CNT-2 shows a value of *E*’ of 2023 MPa at −50 °C (+93% compared to neat PDeF) and 720 MPa at 30 °C (+116%).

The glass transition can be better appreciated from the trend of tanδ (Figure 8b), which shows a peak at 20–30 °C. The tanδ signals of the different compositions have been smoothed and vertically translated to facilitate the comprehension. Figure 8b reports both the experimental data and the smoothed curve, which was used to find the position and intensity of the peaks, reported in Table 5. The peak of tanδ, often considered as the glass transition temperature found in DMA, generally shifts to higher temperatures with CNT addition, which indicates that CNTs hinder the mobility of the amorphous phase of PDeF, as reported elsewhere in the literature [48]. This suggests that the increase in the strain at break that follows CNT addition (see Section 3.3.) is not given by an increased molecular mobility of the amorphous phase, but rather to a decrease in the crystallite size, as already observed in research from the literature [49]. Conversely, the height of the tanδ peak is not remarkably affected by the CNT content.

Another interesting representation of the DMA data is the so-called Cole–Cole plot, which represents the loss modulus as a function of the storage modulus on linear axes. This plot has been reported to give information on the system heterogeneity, secondary relaxations, structural changes after the addition of a second phase, and the thermorheological simplicity or complexity of the system [50,51,52,53]. The Cole–Cole plot is semicircular for homogeneous polymer systems with well-dispersed fillers, while heterogeneous systems show elliptical or imperfect curves [54,55]. The Cole–Cole plots of the nanocomposite films are presented in Figure 8c. The main difference between the PDeF and the nanocomposites is the range of the values of *E*’ and *E*”; the neat PDeF presents a much smaller semicircle as the values of *E*’ and *E*” are much lower than those of the nanocomposites. On the other hand, the qualitative shape of the plots are similar, which indicates that the films are homogeneous and the filler–matrix adhesion is good [55].

### 3.3. Mechanical and Viscoelastic Properties

**Figure 9:**
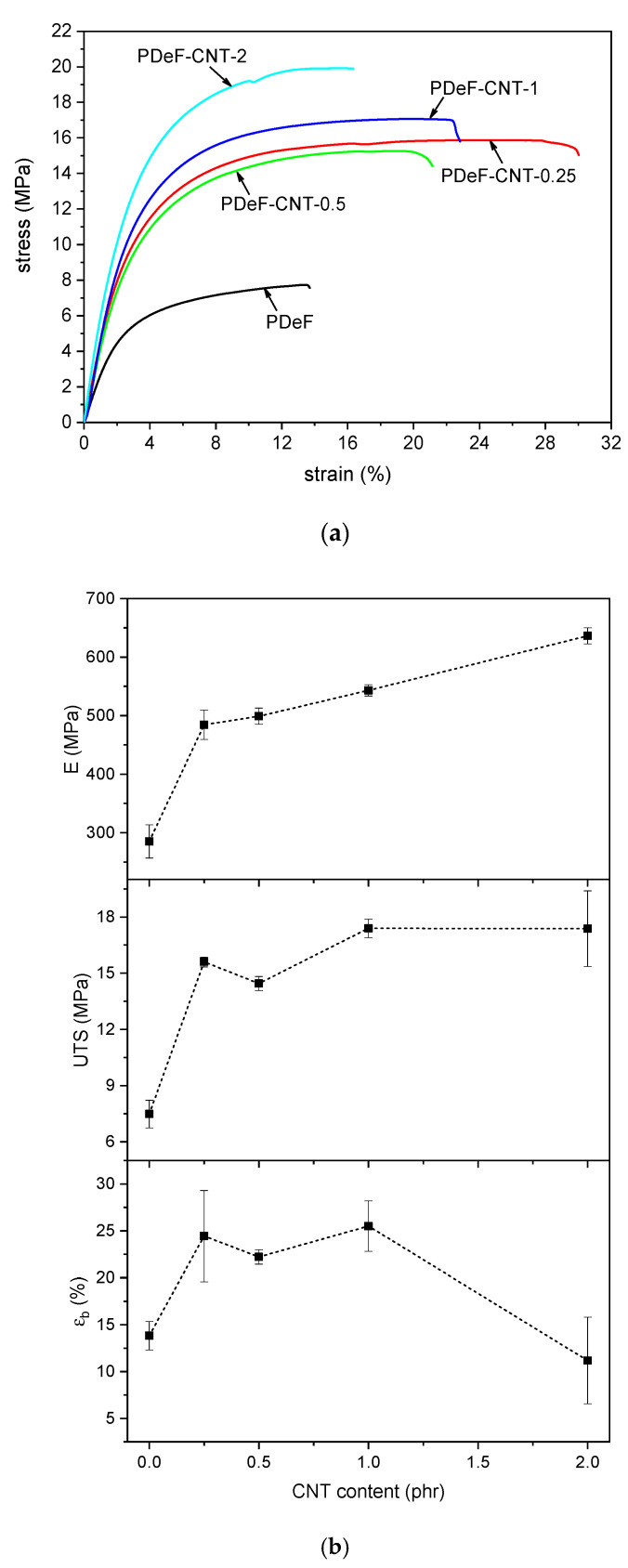
Results of quasi-static tensile tests. (**a**) Representative stress–strain curves obtained in quasi-static tensile tests on the prepared samples; (**b**) trends of elastic modulus (E), ultimate tensile strength (UTS), and strain at break ($\varepsilon_{b}$) as a function of the CNT content.

The addition of CNTs increases the mechanical properties of the host polymer matrix considerably, as proved through quasi-static tensile tests and creep tests. The results of the tensile tests are reported in Figure 9, which shows representative stress–strain curves (Figure 9a) and trends of elastic modulus (E), ultimate tensile strength (UTS), and strain at break ($\varepsilon_{b}$) as a function of the CNT content (Figure 9b). The addition of CNTs considerably increases the elastic modulus, as it rises from 285 ± 28 MPa of the neat PDeF to 636 ± 13 MPa of the sample PDeF-CNT-2, with an increase of 123%. Interestingly, a CNT amount of 0.25 phr already brings a substantial increase in stiffness, as the elastic modulus of the sample PDeF-CNT-0.25 is 484 ± 24 MPa, which is 70% higher than that of the neat PDeF. The same trend can be observed also for the UTS, which ranges from 7.5 ± 0.7 MPa for the neat PDeF, to 15.6 ± 0.2 MPa (+108%) with a CNT content of 0.25 phr, up to 17.3 ± 2.0 MPa (+131%) with a CNT content of 2 wt%. These results confirm the capability of this nanofiller to increase the stiffness and the strength of the host polymer matrices.

Moreover, a small amount of CNTs also promotes an increase in the strain at break. This simultaneous increase in stiffness (and strength) and strain at break has already been observed in the literature for other CNT-reinforced polymers, such as polyethylene [56] and epoxy [57,58]. For the polymer under investigation, the reason could reside in the smaller crystallite dimension induced by the CNTs, as already reported in the literature [49]. XRD analysis seems to point in this direction, but only for a CNT content higher than 0.5 phr, and therefore, further tests are needed to fully understand this aspect. In any case, when the CNT loading increases (2 phr), the stiffening effect becomes predominant and the strain at break falls to values not significantly different from those of the neat PDeF. This result is also probably due to the aggregation of CNTs at high filler content. Better results could be found after improving the CNT disentanglement and dispersion in the polymer matrix. However, these results already confirm that CNTs are an interesting filler for PDeF, and they enhance not only the thermal and functional properties but also the mechanical performance.

**Figure 10:**
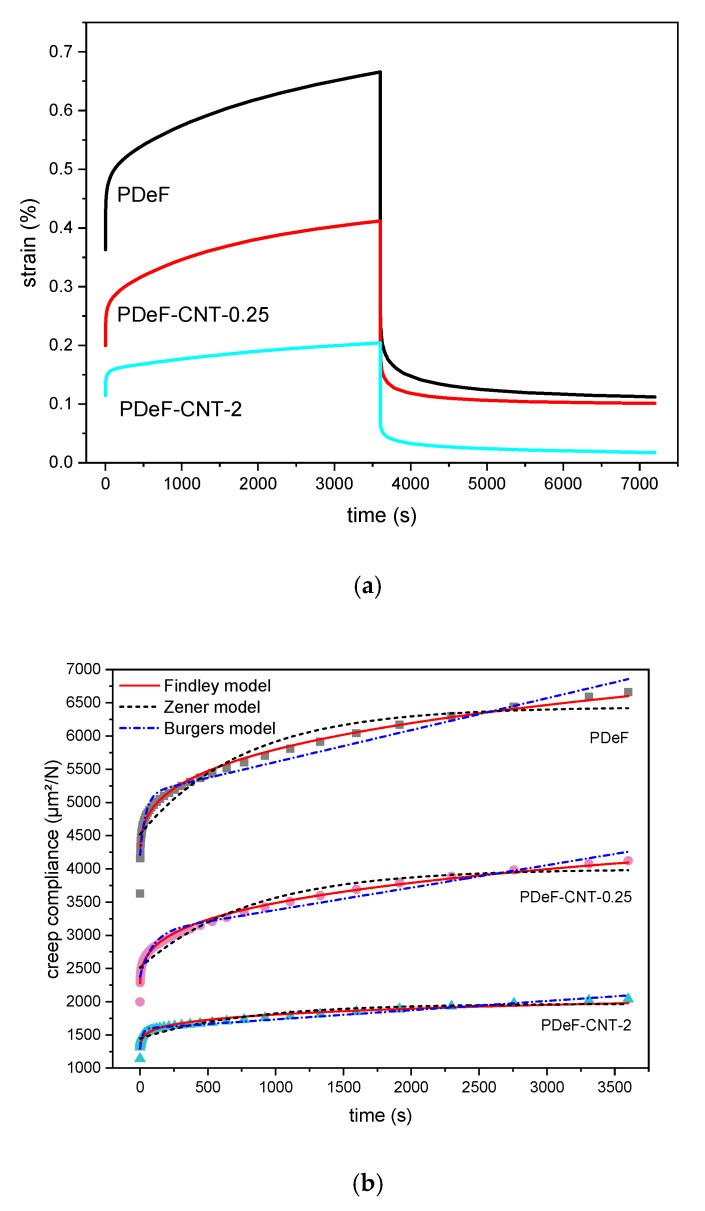
Results of creep-recovery tests. (**a**) Representative curves of strain as a function of time for the samples PDeF, PDeF-CNT-0.25, and PDeF-CNT-2; (**b**) creep compliance as a function of time: Experimental data (symbols) and fitting with the models of Findley, Zener, and Burgers.

Creep and recovery behaviors are of fundamental importance in any application where the material is subjected to prolonged load and must keep dimensional stability. These tests were carried out on samples PDeF, PDeF-CNT-0.25, and PDeF-CNT-2, and the results are reported in Figure 10 and Table 6. The total creep compliance as a function of time during applied stress in the linear viscoelastic region (D(t)) can be measured as the ratio between the measured strain and the constant applied stress. D(t) is given by the sum of an elastic component, which is instantaneous and reversible ($D_{el})$, and a viscoelastic component, which is a function of time ($D_{ve}\left(t\right)$), and is represented in Equation (6) [59]:

(6) $$D\left(t\right)=D_{el}+D_{ve}\left(t\right)$$

The introduction of CNTs clearly improves the creep-recovery behavior of the host matrix. As shown in Table 6, a higher CNT content corresponds to a lower total compliance at the end of the creep stage (D(3600s)), and also to lower values of $D_{el}$, determined at the initial value of deformation, and $D_{ve}\left(3600s\right)$, determined as the difference between D(3600s) and $D_{el}$.

Several equations are available in the literature to model the creep behavior of polymer nanocomposites [60]. Among the most widely used are the models of Findley, Zener, and Burgers. The Findley model is derived by taking the first term of the series expansion of the Kohlrausch–Williams–Watts (KWW) model. The Findley model is presented in Equation (7):

*Dt* = *De* + *k*·*t^n^* (7)

where $D_{e}$ is the initial, elastic compliance, k is a coefficient related to the amplitude of the time-dependent creep, and n is a stress-independent exponent tuning the time dependency of the creep process [61,62]. The experimental data were fitted with the Findley model and the results are presented in Table 6. Again, the initial instantaneous compliance decreases with an increase in the CNT fraction, and so does n, while k is not significantly influenced by the composition. The high values of $R^{2}$ confirm that the Findley model is suitable for representing the experimental results.

To better understand the creep behavior and try to improve the fitting, two additional models were considered, namely the Zener model and the Burgers model [63,64]. The Zener model is a three-element model represented by a spring in series with a Voigt element (a spring and a dashpot in parallel) and is mathematically described in Equation (8) [64]:

(8) $$D\left(t\right)=\frac{1}{E_{1}}+\frac{1}{E_{2}}\cdot \left[1-\exp\left(-t\cdot \frac{E_{2}}{\eta }\right)\right]$$

where $E_{1}$ and $E_{2}$ are the stiffness of the isolated spring and of the spring of the Voigt element, respectively, and η is the viscosity of the dashpot of the Voigt element. The Burgers model is a four-element model corresponding to a Maxwell element (a spring and a dashpot in series) in series with a Voigt element and is mathematically represented in Equation (9) [64]:

(9) $$D\left(t\right)=\frac{1}{E_{1}}+\frac{1}{E_{2}}\cdot \left[1-\exp\left(-t\cdot \frac{E_{2}}{\eta_{2}}\right)\right]+\frac{t}{\eta_{1}}$$

where $E_{1}$ and $E_{2}$ are the stiffness of the springs of the Maxwell and Voigt elements, respectively, and $\eta_{1}$ and $\eta_{2}$ are the viscosity of the dashpot of the Maxwell and Voigt elements, respectively. The Burgers model can also be seen as the Zener model with an additional dashpot in series. The results of the fitting with these two additional models is reported in Table 6.

From the data of $R^{2}$ and from the curves in Figure 10b, it can be observed that the Zener model does not predict the experimental behavior accurately, especially in the initial stages of creep, and it also underestimates the final total creep compliance. An improved fitting is obtained with the Burgers model, although the Findley model outperforms both the other two models in fitting capacity, at least for the samples PDeF and PDeF-CNT-0.25. For the sample with the highest amount of CNTs, the Burgers model gives the best results, as the other two models tend to underestimate the final total compliance and fail in predicting the initial creep behavior accurately.

### 3.4. Electrical Properties

**Figure 11:**
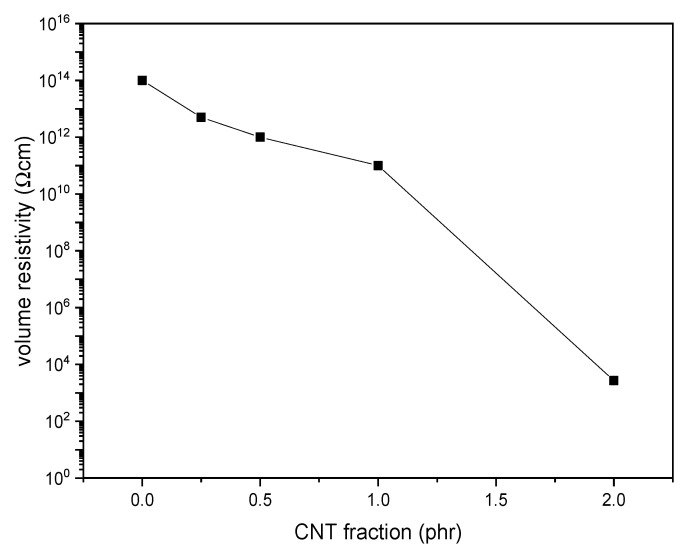
Volume resistivity of the prepared samples as a function of the carbon nanotube (CNT) content.

Figure 11 shows the results of the electrical resistivity tests on the prepared films. The volume resistivity (*ρ*) of the neat PDeF is 1.3·10^14^ Ω·cm, and the resistivity decreases with the CNT fraction. The decrease is modest until a CNT loading of 1 phr, while the material becomes markedly more conductive with a CNT content of 2 phr. Films for packaging and electronics are often classified according to the Standard ANSI/EIA-541, “Packaging Materials Standards for ESD sensitive Items.” The standard divides the materials into insulative (*ρ* > 10^11^ Ω·cm), dissipative (10^4^ Ω·cm < *ρ* < 10^11^ Ω·cm), and conductive (*ρ* < 10^4^ Ω·cm) and classifies as antistatic the materials that are either dissipative or conductive. According to this classification, the films investigated in the present work are insulative with a CNT loading lower than 1 phr, dissipative (and antistatic) with a CNT loading equal to 1 phr, and conductive (and antistatic) with a CNT loading equal to 2 phr.

## 4. Conclusions

The present work investigated the microstructural, thermo-mechanical, and electrical properties of CNT-based nanocomposites of a promising biobased polymer, i.e., poly(decylene furanoate), PDeF. The microstructural characterization evidenced that the fracture surface became smoother and more homogeneous with a small fraction of CNTs, and that the production process allowed a good debundling and dispersion of CNTs in the matrix to be achieved, even though some aggregates were still observable. DSC analysis showed that CNTs acted as nucleating agents for PDeF, and the crystallinity degree in the first DSC heating scan increased from 43.2% of PDeF to 55.0% of PDeF-CNT-2. A similar trend of crystallinity was evidenced by XRD with Rietveld analysis with a three-phase model. Additionally, the crystallization temperature in the cooling scan increased from 68.4 °C of PDeF to 91.7 °C of PDeF-CNT-2, which was another piece of evidence of the nucleating action promoted by CNTs and could be a signal of an increased thermal conductivity. DMA tests showed that the storage modulus increased considerably with CNT addition and the peak of tanδ, considered as a measurement of the $T_{g}$, generally shifted to higher temperatures, which implied that CNTs hindered the mobility of the amorphous phase. Moreover, the Cole–Cole plots of the nanocomposites were qualitatively similar to that of the neat PDeF, which was a signal of the homogeneity of the systems and of a good filler–matrix adhesion. CNTs also contributed to the mechanical performance of the bioderived polymer, which showed an elastic modulus (285 ± 28 MPa) and a mechanical strength (7.5 ± 0.7 MPa) similar to those of LDPE. The elastic modulus increased up to 123% and the stress at break up to 131%. Surprisingly, the strain at break also increased for a small amount of CNTs (+71% with a CNT content of 0.25 phr), which was probably the consequence of a more homogeneous microstructure. The long-term mechanical performance was also improved upon CNT addition, as the creep compliance decreased considerably, which was observed both for the elastic and for the viscoelastic component. Finally, CNTs also promoted a decrease in the electrical resistivity, and the films became electrically dissipative for a CNT content of 1 phr and conductive for a CNT content of 2 phr. This study contributed to highlight the properties of the bioderived furan-based polymer PDeF and evidenced the potential of CNTs as a filler for this matrix, as they enhanced the mechanical and the functional properties of the resulting materials.

## Figures and Tables

**Table 1:** List of the prepared samples with nominal weight compositions.

Sample	PDeF (phr)	CNT (phr)
PDeF	100	0
PDeF-CNT-0.25	100	0.25
PDeF-CNT-0.5	100	0.5
PDeF-CNT-1	100	1
PDeF-CNT-2	100	2

PDeF = poly(decylene furanoate); CNT = carbon nanotubes; phr = parts per hundred resin (g per 100 g of polymer).

**Table 2:** Semi-quantitative results as obtained from Rietveld modeling compared with the crystallinity degree calculated from integrated intensities ratio. Uncertainties for phase volume fractions are reported as the last significant digit.

Sample	$R_{wp}$	PDeF Crystalline (vol%)	PDeF Nanocryst (vol%)	PDeF Amorph (vol%)	$X_{c}$ (Rietveld) (%)	$X_{c}$ (Int. Ratio) (%)
PDeF	9.54	28.2(3)	29.8(1)	41.9(6)	58.0	43.5
PDeF-CNT-0.25	9.52	37.8(3)	22.9(9)	39.1(9)	60.8	54.7
PDeF-CNT-0.5	9.82	37.5(3)	21.8(6)	40.5(9)	59.4	52.7
PDeF-CNT-1.0	8.68	30.3(2)	30.1(6)	39.5(1)	60.5	53.6
PDeF-CNT-2.0	7.36	17.6(1)	45.7(3)	36.6(6)	63.3	56.7

$R_{wp}$ = figure of merit of Rietveld analysis; $X_{c}$ = degree of crystallinity.

**Table 3:** Main results of differential scanning calorimetry (DSC) tests on the prepared samples.

Sample	$T_{m\_1}$ (°C)	$∆H_{m_{1}}$ (J/g)	${Χ}_{c}$ (%)	$T_{c}$ (°C)	$∆H_{c}$ (J/g)	$T_{g\_2}$ (°C)	$T_{m\_2}$ (°C)	$∆H_{m\_2}$ (J/g)
PDeF	110.2	66.2	43.2	68.4	49.4	−4.0	110.4	47.9
PDeF-CNT-0.25	113.9	74.8	49.0	88.7	54.3	−8.5	111.1	58.7
PDeF-CNT-0.5	114.3	78.5	51.6	89.1	53.9	−10.8	111.3	58.6
PDeF-CNT-1	113.7	70.9	46.8	91.1	55.0	−4.2	111.9	59.4
PDeF-CNT-2	115.5	82.6	55.0	91.7	57.7	−2.0	111.9	66.5

$T_{m\_1}$ = melting temperature (first heating scan); $∆H_{m\_1}$ = melting enthalpy (first heating scan); ${Χ}_{c}$ = crystallinity degree (first heating scan); $T_{c}$ = crystallization temperature; $∆H_{c}$ = crystallization enthalpy; $T_{g\_2}$ = glass transition temperature (second heating scan); $T_{m\_2}$ = melting temperature (second heating scan); $∆H_{m\_2}$ = melting enthalpy (second heating scan).

**Table 4:** Main results of TGA tests on the prepared samples.

	$T_{1\%}$ (°C)	$T_{3\%}$ (°C)	$T_{5\%}$ (°C)	$T_{d}$ (°C)
PDeF	220.7	311.4	340.4	393.0
PDeF-CNT-0.25	198.4	289.7	328.1	388.4
PDeF-CNT-0.5	152.6	271.9	316.5	391.0
PDeF-CNT-1	141.0	264.3	319.0	390.0
PDeF-CNT-2	152.9	275.5	328.7	388.9

$T_{1\%}$, $T_{3\%},\;T_{5\%}$ = temperatures corresponding to mass losses of 1 wt%, 3 wt%, and 5 wt%; $T_{d}$ = degradation temperature, intended as the peak of the mass loss derivative signal.

**Table 5:** Results of dynamic-mechanical analysis (DMA) on the prepared samples. Values of *E*’ at −50 °C and 30 °C, intensity of tan *δ* peak, and relative position (regarded as the glass transition temperature).

Sample	*E*’ (−50 °C) (MPa)	*E*’ (30 °C) (MPa)	tan δ Peak Intensity	tan δ Peak Position (°C)
PDeF	1049	333	0.107	23.4
PDeF-CNT-0.25	1904	682	0.113	23.9
PDeF-CNT-0.5	1957	750	0.105	30.7
PDeF-CNT-1	2145	770	0.115	26.9
PDeF-CNT-2	2023	720	0.101	27.0

**Table 6:** Results of the creep test for the samples PDeF, PDeF-CNT-0.25, and PDeF-CNT-2, and fitting parameters with the models of Findley, Zener, and Burger.

		PDeF	PDeF-CNT-0.25	PDeF-CNT-2
Experimental	D(3600s) (µm^2^/N)	6659	4119	2040
	$D_{el}$ (µm^2^/N)	3628	1996	1141
	$D_{ve}\left(3600s\right)$ (µm^2^/N)	3031	2123	899
Findley model	$D_{e}$ (µm^2^/N)	4032 ± 52	2069 ± 58	1065 ± 107
	k (µm^2^/(N·s))	232 ± 30	204 ± 35	239 ± 90
	n (-)	0.293 ± 0.014	0.280 ± 0.019	0.163 ± 0.030
	*R^2^*	0.994	0.989	0.960
Zener model	$E_{1}$ (MPa)	221 ± 2	398 ± 5	691 ± 8
	$E_{2}$ (MPa)	520 ± 33	673 ± 35	1910 ± 160
	η (GPa·s)	400 ± 60	546 ± 65	1500 ± 280
	*R^2^*	0.911	0.941	0.860
Burgers model	$E_{1}$ (MPa)	239 ± 4	422 ± 6	785 ± 14
	$E_{2}$ (MPa)	1060 ± 81	1490 ± 110	3110 ± 233
	$\eta_{1}$ (GPa·s)	2084 ± 132	2950 ± 209	7202 ± 380
	$\eta_{2}$ (GPa·s)	44 ± 10	135 ± 28	53 ± 12
	*R^2^*	0.964	0.970	0.966
